# Modern Longevity

**Published:** 1854-04

**Authors:** 


### MODERN LONGEVITY.

The following are the names of persons who have died within a few weeks past:

| Name. | Place. | Age. |
|---|---|---|
| Capt. A. Partridge | Connecticut | 70 |
| Thomas Gascoigne | ——— | 70 |
| W. W. Groesbeck | New York | 70 |
| Thomas Banks | Virginia | 70 |
| Joseph Trotter | Philadelphia | 71 |
| Thomas Gould | Illinois | 71 |
| Hon. John Gebhard | New York | 72 |
| Charles Vezin | Philadelphia | 73 |
| Dr. Samuel Carter | Brooklyn | 75 |
| Samuel Brooks | Philadelphia | 75 |
| William Wilson | ——— | 75 |
| John Armstrong | Indiana | 77 |
| Jacob Dunton | Philadelphia | 80 |
| Ann Mather | Pennsylvania | 80 |
| Sarah Ward | Sing Sing | 81 |
| William Gardner | England | 83 |
| Archibald Davidson | New York | 83 |
| Maj. Sam. Rosseter | ——— | 85 |
| Rev. William Jay | England | 85 |
| Mary T. Dickinson | Philadelphia | 85 |
| C. C. Watson | Philadelphia | 87 |
| Thomas H. Perkins | Massachusetts | 89 |
| Rev. David Compost | New York | 90 |
| Abraham Teshune | New York | 94 |
| Andrew Davis | New York | 93 |
| Rachel Campbell | Pennsylvania | 96 |

It is worthy of remark, that in every instance of long life given above, of names collected in half-an hour from a bundle of newspapers lying on the floor, the subject was either a man of acknowledged piety or was possessed of high and honorable business capacities; thus offering the premium of a good old age, to all those who choose to live a life of piety and active honorable enterprise and industry. And in view of the following examples of effective working old age, ought not every young clergyman who is prematurely disabled, to investigate the cause, and instead of laying it on the Lord, and calling it a mysterious dispensation of Providence, begin to fear lest the fault lie at his own door, in unwarranted indulgencies or unwise habits of life.

* * * * * * Dr. Van Oven, in his work "On the Decline of Life in Health and Disease," comes to the conclusion that a hundred years and upwards, even considerably upwards, is the term which man *ought*, by care and prudence, to attain.

*The Old Colony Memorial* (of Plymouth) says—"Dr. James Kendall, of this place, preached to his people last Sabbath, from the text—'Having therefore obtained help of God, I continue unto this day,' &c. Dr. Kendall is in his eighty-sixth year, and was settled over his people in the year 1800—making a ministry of fifty-four years."

Death of Rev. William Jay, England.—The last steamer brought the intelligence of the death of this venerable man, who departed this life at Bath, Dec. 27th, 1853, in the 85th year of his age. He was the author of the "Morning and Evening Exercises," which bear his name, and which has profited so many thousands of Christians. He commenced preaching at the age of sixteen, at Surrey Chapel, but his regular ministry was confined to Bath, and was never interrupted until he resigned a short time since. He has gone to his grave like a shock of corn, fully ripe. Few men have lived to accomplish so much good, or to leave behind them a name so honored and precious.

The venerable Rev. David Comfort departed this life on the 28th ult., in the 90th year of his age, and the fifty-fourth of his ministry, fifty of which were spent as the pastor of one flock, that of Kingston, N. J.

Rev. Father Nott, of Franklin, Connecticut, died within a year or two aged eighty-five years, and up to the age of eighty years performed his pastoral duties with ability and energy, with a mind active and wakeful to the last.

